# Decision-tree model for predicting outcomes after out-of-hospital cardiac arrest in the emergency department

**DOI:** 10.1186/cc12812

**Published:** 2013-07-11

**Authors:** Yoshikazu Goto, Tetsuo Maeda, Yumiko Goto

**Affiliations:** 1 Section of Emergency Medicine, Kanazawa University Hospital, 13-1 Takaramachi, Kanazawa 920-8641, Japan; 2 Department of Cardiology, Yawata Medical Center, 12-7 I Yawata, Komatsu 923-8551, Japan

**Keywords:** cardiac arrest, cardiopulmonary resuscitation, emergency department, outcome, prediction model

## Abstract

**Introduction:**

Estimation of outcomes in patients after out-of-hospital cardiac arrest (OHCA) soon after arrival at the hospital may help clinicians guide in-hospital strategies, particularly in the emergency department. This study aimed to develop a simple and generally applicable bedside model for predicting outcomes after cardiac arrest.

**Methods:**

We analyzed data for 390,226 adult patients who had undergone OHCA, from a prospectively recorded nationwide Utstein-style Japanese database for 2005 through 2009. The primary end point was survival with favorable neurologic outcome (cerebral performance category (CPC) scale, categories 1 to 2 [CPC 1 to 2]) at 1 month. The secondary end point was survival at 1 month. We developed a decision-tree prediction model by using data from a 4-year period (2005 through 2008, *n *= 307,896), with validation by using external data from 2009 (*n *= 82,330).

**Results:**

Recursive partitioning analysis of the development cohort for 10 predictors indicated that the best single predictor for survival and CPC 1 to 2 was shockable initial rhythm. The next predictors for patients with shockable initial rhythm were age (<70 years) followed by witnessed arrest and age (>70 years) followed by arrest witnessed by emergency medical services (EMS) personnel. For patients with unshockable initial rhythm, the next best predictor was witnessed arrest. A simple decision-tree prediction mode permitted stratification into four prediction groups: good, moderately good, poor, and absolutely poor. This model identified patient groups with a range from 1.2% to 30.2% for survival and from 0.3% to 23.2% for CPC 1 to 2 probabilities. Similar results were observed when this model was applied to the validation cohort.

**Conclusions:**

On the basis of a decision-tree prediction model using four prehospital variables (shockable initial rhythm, age, witnessed arrest, and witnessed by EMS personnel), OHCA patients can be readily stratified into the four groups (good, moderately good, poor, and absolutely poor) that help predict both survival at 1 month and survival with favorable neurologic outcome at 1 month. This simple prediction model may provide clinicians with a practical bedside tool for the OHCA patient's stratification in the emergency department.

## Introduction

In Japan, approximately 100,000 out-of-hospital cardiac arrests (OHCAs) occur annually, and nationwide improvements in favorable neurologic outcomes after cardiac arrest have been observed after connecting the links in the "chain of survival" [1,2]. However, the outcomes of very elderly patients have not improved and are generally dismal, irrespective of the origin of the OHCA [2].

Patient outcomes after cardiac arrest are associated with a multitude of variables, including age, comorbidities, initial recorded cardiac rhythm, and other circumstances related to cardiac arrest, such as the time to return of spontaneous circulation (ROSC) [3,4]. The more-fascinating but controversial aspect of outcome prediction is the possibility of helping guide decision making and risk assessment for individual patients [4]. By predicting which treatment strategies will be futile for an individual, human suffering and costs could be reduced while increasing the capacity for treating other critically ill patients [4].

Multivariate analyses have identified factors that have enabled the development of sophisticated equations and scoring models with the ability to predict outcomes after OHCA [5-9]. However, the comparability of different cohorts has been questioned. In addition, direct outcome comparisons may be hindered further by differing definitions of inclusion and exclusion criteria [9]. Therefore, implementation of such outcome-prediction equations and scores in research and clinical practice has been slow [4].

The more crucial aspect of these predictions is the lack of stratification of prehospital factors for OHCA patients. A useful way to think of predictors is to consider patient factors (age, comorbid illnesses, and so on), event factors (witnessed, public versus private, and so on), emergency medical services (EMS) factors (response times, crew type), and treatment factors. A simple and reliable prediction model for patients with OHCA may help clinicians guide in-hospital strategies, particularly in the emergency department (ED).

The purpose of this study was to develop a simple and generally applicable prediction model for adult patients after nontraumatic OHCA.

## Materials and methods

### Study design and data source

The present investigation was a nationwide population-based observational study of all adult patients (age, >18 years) for whom resuscitation had been attempted after nontraumatic OHCA in Japan from January 1, 2005, to December 31, 2009. Cardiac arrest was defined as the cessation of cardiac mechanical activities, as confirmed by the absence of signs of circulation [1]. This study was approved by the Ethical Committee of Kanazawa University. The requirement of written informed consent was waived.

### Emergency medical services system in Japan

Japan has approximately 127 million residents in an area of 378,000 km^2^, approximately two thirds of which is uninhabited mountainous terrain [10]. The Fire and Disaster Management Agency (FDMA) of Japan supervises only the EMS system nationwide [11]. The EMS system is operated by each local fire station. The toll-free telephone emergency number 1-1-9 is used to call for ambulance assistance from anywhere in Japan. Generally, an ambulance crew includes three EMS staff members, including at least one emergency life-saving technician (ELST) [1,10]. Under the online medical control, ELSTs are allowed to use several resuscitation methods, including semiautomated external defibrillators, insertion of a supraglottic airway device (laryngeal mask airway, laryngeal tube, and esophagotracheal twin-lumen airway device), insertion of a peripheral intravenous line, and administration of Ringer lactate solution [1]. Only specially trained ELSTs can perform endotracheal intubation and administration of intravenous adrenalin under medical control direction. The termination-of-resuscitation rule for EMS personnel has been developed and commonly applied at emergency scenes worldwide [3,12,13]. However, EMS personnel in Japan are legally prohibited from terminating resuscitation in the field. Most OHCA patients undergo cardiopulmonary resuscitation (CPR) by EMS providers and are transported to hospitals, except in cases in which fatality is certain [10,11].

### Data collection and quality control

The FDMA launched a prospective population-based observational study involving all OHCA victims who received EMS in Japan [1]. EMS personnel at each center recorded data for OHCA victims with the cooperation of the physician in charge of the victims, by using an Utstein-style template [14]. All the data were transferred and stored in the nationwide database developed by the FDMA for public use. We analyzed this database with the permission of the FDMA. The FDMA provided all the anonymous data to our research group.

The main items included in the database were as follows: sex, age, causes of arrest (presumed cardiac etiology or not), bystander-witness status, bystander CPR with or without automated external defibrillator (AED) use, initial identified cardiac rhythm, bystander category (that is, if a bystander was present, then whether the bystander was a layperson or EMS personnel), ROSC before arrival at the hospital, time of the emergency call, time of vehicle arrival at the scene, time of ROSC, time of vehicle arrival at the hospital, 1-month survival, and neurologic outcome at 1 month after cardiac arrest. The neurologic outcome was defined in terms of the Cerebral Performance Category (CPC) scale: category 1, good cerebral performance, category 2, moderate cerebral disability, category 3, severe cerebral disability, category 4, coma or vegetative state and category 5, death [14]. This CPC categorization was determined by the physicians in charge. The call-response time interval was calculated as the time from the emergency call to the time of vehicle arrival at the scene [11]. The call-to-hospital arrival time interval was calculated as the time from the emergency call to the time of vehicle arrival at the hospital.

### End points

The primary study end point was survival at 1 month with favorable neurologic outcome, which was defined as a CPC of 1 or 2 [14]. The secondary end point was survival at 1 month.

### Statistical analysis

We selected 10 prehospital variables for developing a prediction model. The 10 explanatory prehospital variables that were related to patient characteristics and resuscitation were as follows: age, gender (male or female), witnessed arrest (yes or no), arrest witnessed by EMS personnel (yes or no), bystander CPR (yes or no), cardiac cause (yes or no), initial cardiac rhythm recorded (shockable or not), prehospital AED administration (yes or no), call-to-response time interval, and call-to-hospital arrival time interval. We treated the seven variables as dichotomous variables.

As a recursive partitioning analysis may be more suitable than logistic regression when the intent is to classify one outcome at the expense of another [15], we performed recursive portioning analysis to develop a decision-tree model for outcome prediction. Recursive partitioning analysis creates a branching decision tree by dividing the patient population into subgroups according to the results of analysis of the relation between proportions of outcomes after OHCA and prehospital variables. The recursive portioning was conducted by using the maximized entropy index [16-18]. Ten-fold cross-validation was used to assess the predictive ability of the decision-tree model.

Statistical analyses were performed by using the Wilcoxon and Kruskal-Wallis tests for continuous variables and the χ^2 ^tests for categoric variables. Continuous variables are expressed in terms of mean and standard deviation (SD) values. Categoric variables are expressed in terms of percentages. As an estimate of effect size and variability, we report odds ratios (ORs) with 95% confidence intervals (CIs). We assessed overall model discrimination by using the area under the receiver operating characteristic curve (AUC). All statistical analyses were performed with the JMP statistical package version 9 (SAS Institute Inc., Cary, NC, USA). All tests were two tailed, and a value of *P *< 0.05 was considered statistically significant.

## Results

**Figure 1:**
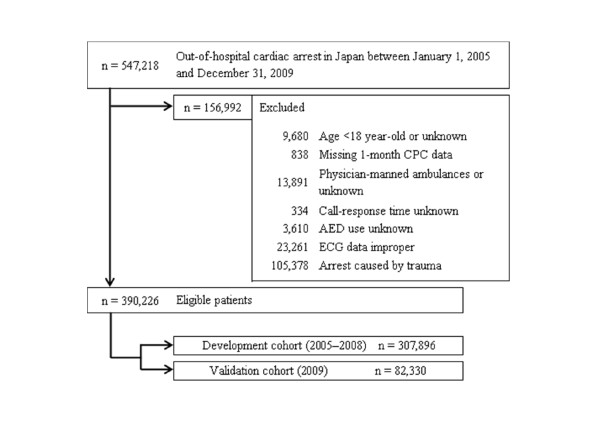
**Study profile with selection of participants**. AED, automated external defibrillator; CPC, cerebral performance category; ECG, electrocardiogram.

During the 5-year study period, 541,218 patients were documented in the database. We considered 390,226 (72.1%) patients eligible for enrolment into this study. Figure 1 shows a flow diagram depicting the inclusion/exclusion criteria for subjects in the present study. We developed a decision-tree model by using data from a 4-year period (2005 through 2008; *n *= 307,896), with validation using external data from 2009 (*n *= 82,330). The characteristics of all the subjects and the results of univariate analysis between two cohorts used for the development and validation of the models are shown in Table 1. The overall 1-month survival and favorable neurologic outcome (CPC 1 to 2) rates were 4.2% and 2.0%, respectively. The prehospital variables significantly differed between the two cohorts, with the exception of the ratios of witnessed arrest, shockable initial rhythm, and prehospital AED administration. Although an older patient age and longer vehicle traveling time were noted in the validation cohort, significant increases were identified in the ratios of prehospital ROSC, 1-month favorable neurologic outcome, and 1-month survival.

**Table 1:** Baseline characteristics and outcomes of the study patients

Characteristics	All patients		Development cohort (2005 to 2008)		Validation cohort (2009)		*P *value
		
	*n *= 390,226 (100%)		*n *= 307,896 (78.9%)		*n *= 82,330 (21.1%)		
Age, years, mean ± SD	74.8	±14.7	74.5	±14.7	75.6	±14.5	<0.0001
Male, *n *(%)	225,152	(57.7%)	178,165	(57.9%)	46,987	(57.1%)	<0.0001
Witnessed arrest, *n *(%)	149,701	(38.4%)	117,986	(38.3%)	31,715	(38.5%)	0.291
Arrest witnessed by EMS personnel, *n *(%)	18,581	(4.8%)	14,321	(4.7%)	4,260	(5.2%)	<0.0001
Bystander CPR, *n *(%)	165,412	(42.4%)	123,980	(40.3%)	41,432	(50.3%)	<0.0001
Presumed cardiac etiology, *n *(%)	276,182	(70.8%)	216,241	(70.2%)	59,941	(72.8%)	<0.0001
Shockable initial rhythm, *n *(%)	36,594	(9.4%)	28,745	(9.3%)	7,849	(9.5%)	0.084
Prehospital AED administration (actual shock delivery)	49,556	(12.7%)	39,145	(12.7%)	10,411	(12.7%)	0.601
Call-response time interval, minutes, mean ± SD	7.24	±3.73	7.17	±3.74	7.49	±3.66	<0.0001
Call-hospital arrival time interval, minutes, mean ± SD	29.9	±9.9	29.7	±9.8	30.5	±10.0	<0.0001
Prehospital ROSC	20,547	(5.3%)	15,361	(5.0%)	5,186	(6.3%)	<0.0001
Outcome 1 month after cardiac arrest							
Survival, *n *(%)	16,332	(4.2%)	12,514	(4.1%)	3,818	(4.6%)	<0.0001
Favorable neurologic outcome (CPC = 1 to 2), *n *(%)	7,768	(2.0%)	5,777	(1.9%)	1991	(2.4%)	<0.0001

AED, automated external defibrillator; CPC, cerebral performance category; CPR, cardiopulmonary resuscitation; EMS, emergency medical services; ROSC, return of spontaneous circulation; SD, standard deviation.

**Figure 2:**
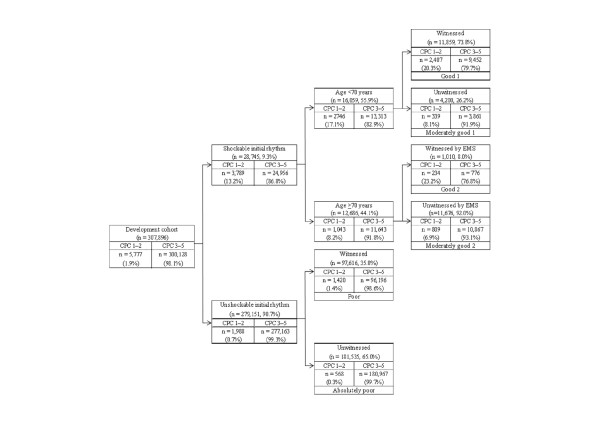
**Decision-tree model of recursive partitioning analysis for predicting favorable neurologic outcomes at 1 month after out-of-hospital cardiac arrest and prediction groups in the development cohort**. CPC, cerebral performance category; EMS, emergency medical services.

Figure 2 depicts the final decision-tree model of the recursive partitioning analysis for predicting favorable neurologic outcome at 1 month in the development cohort. The analysis identified shockable initial rhythm as the best single discriminating factor between CPC 1 to 2 and CPC 3 to 5. The next-best predictor of neurologic outcome in the shockable initial rhythm node was age, at a discrimination level of younger than 70 years. For the node of patients with shockable initial rhythm and the age of younger than 70 years, witnessed arrest provided additional prognostic value. For the node of patients with shockable initial rhythm and the age of 70 years or older, arrest witnessed by EMS personnel provided additional value.

In the unshockable initial rhythm node, the next best predictor of neurologic outcome was witnessed arrest. These branch points permitted stratification into six prediction groups: good 1 (shockable initial rhythm, age <70 years, and witnessed arrest); moderately good 1 (shockable initial rhythm, age <70 years, and unwitnessed arrest); good 2 (shockable initial rhythm, age >70 years, and witnessed by EMS personnel); moderately good 2 (shockable initial rhythm, age >70 years, and unwitnessed by EMS personnel); poor (unshockable initial rhythm and witnessed arrest); and absolutely poor (unshockable initial rhythm and unwitnessed arrest). The prediction rates of CPC 1 to 2 ranged from 0.3% to 23.2% in the absolutely poor and good groups.

The decision-tree model generated by the recursive partitioning analysis was tested for its ability to stratify patients in the validation cohort. The AUCs for this model in the cohorts for development and validation were 0.85 (95% CI, 0.85 to 0.86) and 0.88 (95% CI, 0.87 to 0.89), respectively. Table 2 summarizes the definition of prediction groups for OHCA by using four prehospital factors. In the poor and absolutely poor groups, only two factors (shockable initial rhythm or not and witnessed arrest or not) could stratify patients after OHCA.

**Table 2:** Definition of prediction groups for out-of-hospital cardiac arrest

Prediction groups		Prehospital factors			
		
		Shockable initial rhythm	Age (years)	Witnessed arrest	Witnessed by EMS personnel
Good	1	Yes	<70	Yes	
	2	Yes	>71		Yes
Moderately good	1	Yes	<70	No	
	2	Yes	>71		No
Poor		No		Yes	
Absolutely poor		No		No	

**Figure 3:**
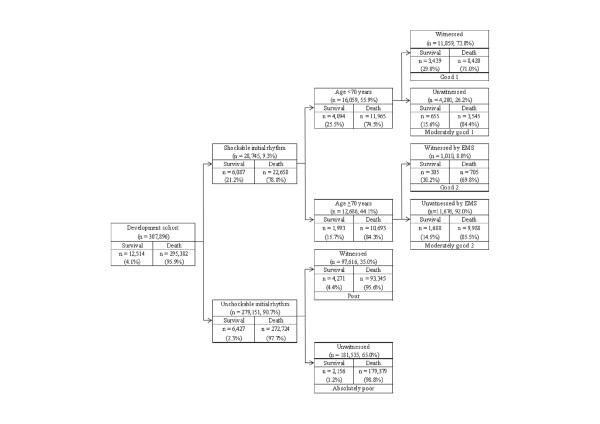
**Decision-tree model of recursive partitioning analysis for predicting survival at 1 month after out-of-hospital cardiac arrest and prediction groups in the development cohort**. EMS, emergency medical services.

The decision tree generated by analysis of the development cohort for 1-month CPC 1 to 2 was tested for its ability to stratify patients for 1-month survival (Figure 3). The prediction rates of survival ranged from 1.2% to 30.2% in the absolutely poor and good groups. The AUCs for this model in the cohorts for development and validation were 0.79 (95% CI, 0.78 to 0.79) and 0.81 (95% CI, 0.80 to 0.82), respectively.

## Discussion

The present analysis of more than 390,000 adult patients with nontraumatic OHCA in Japan demonstrates that neurologic outcomes and survival at 1 month after cardiac arrest can be reliably estimated by using four routinely available prehospital variables, that is, shockable initial rhythm, age, witnessed arrest, and witnessed by EMS personnel, obtained in the ED with the appropriate values of AUC. Overall, the 1-month favorable neurologic outcome (CPC 1 to 2) rate was 2.0%, although this rate varied more than 70-fold (from 0.3% to 23.2%) based on our newly developed decision-tree model. The prediction model should aid medical decision making for patients after OHCA in the ED. Patients judged to be in the good group (Table 2, Figures 2 and 3) may receive higher-level care, such as extracorporeal membrane oxygenation, intraarrest percutaneous intervention [19], and targeted temperature management [20], whereas patients estimated to be in the absolutely poor group should be considered for termination of resuscitation after advanced life support, in accordance with the appropriate guidelines [21,22].

Criteria for the decision to withdraw CPR or perform aggressive mechanical cardiac support in the ED remain elusive. A simple user-friendly score or model to predict patient outcomes before withholding any drugs or CPR attempts under poor baseline conditions in the ED has not yet been fully developed. The German Resuscitation Registry Group developed the ROSC after cardiac arrest (RACA) score [9] for predicting ROSC (defined as a palpable pulse for >20 seconds), which is based on an equation involving 15 prehospital variables. Although the RACA score enables the prediction of initial resuscitation success, it was developed to serve as an instrument for adjusting different conditions [9]. Multiple evaluations of patients after successful CPR have demonstrated the association between clinical outcomes and indices of prehospital and in-hospital factors [5-8,23] at various stages. The OHCA score [5] (evaluated in the intensive care unit (ICU)), initial rhythm, estimated no-flow and low-flow interval, blood lactate, and creatinine levels were used to build a continuous severity score, with AUCs of 0.82 in the development cohort and 0.88 in the validation cohort predicting good neurologic recovery. A model based on four selected indicators (age, time from arrest to ROSC, presence of prehospital ROSC, and shockable initial rhythm or conversion to shockable rhythm) showed a high predictive value for favorable outcome, with an AUC of 0.87 in the external-validation cohort [6].

In the early illness severity score [7] (evaluated within 6 hours of arrest), four distinct categories of post-cardiac arrest were identified by using the serial organ-function assessment score and complete outline of the unresponsiveness score at the time of ICU arrival.

More recently, the 5-R score [8] was developed to aid decision making in targeted temperature management in the ED, based on five independent variables: initial rhythm, arrest-to-first CPR attempt interval, arrest-to-ROSC interval, absence of rearrest, and recovery of the pupillary light reflex.

A significant disadvantage of multivariable-generated prediction models is their complexity. Because of the number of variables and complex mathematical functions involved, a calculator is frequently required to determine the score, thereby making these models impractical for bedside use, especially in the ED. Even when converted to point scores, the tools derived from a multivariate model still require a reference monogram to convert the point scores to risk estimates [24]. Contrary to these sophisticated prediction models, our current decision-tree model is a simple and generally applicable stratification for evaluating patients after OHCA.

In general, prediction models or scores are developed under the same conditions for basic characteristics between the development and validation cohorts [5-9]. This may cause inaccurate predictions for different cohorts, or nonaverage cohorts. In this study, several basic characteristics, such as age, gender, and frequency of bystander CPR, were significantly different between the development and validation cohorts. Significant outcome improvements in the validation cohort (data from 2009) were also seen, compared with those in the development cohort (data from 2005 to 2008). Even in such conditions, our decision-tree model for CPC 1 to 2 had an appropriate AUC of 0.88 (95% CI, 0.87 to 0.89) in the validation cohort. These results indicate that our decision-tree model might be applicable for other countries with different EMS systems.

Although prehospital ROSC is the single most important predictor of outcomes thus far [3], we developed our prediction model without using prehospital ROSC as a covariate factor because it is considered to be involved in the causal pathway [25]. Our final decision-tree model did not include any time variables. Any prediction model using time intervals may be difficult to apply in practice during CPR in the ED. The recursive partitioning analysis applied in the present study can detect interactions between variables [18] and yields an easily available stratification model at the bedside. This method was applied widely for generating clinical risk-stratification schemes for cardiac [24], oncologic [26], and infectious disorders [27].

Implementation of mild therapeutic hypothermia [28,29] and the aggressive management [30] of postresuscitation syndrome significantly improved outcomes after OHCA [31]. These recent findings may modify the accuracy of prognosis. A multimodality prediction approach, including neurologic examination, electroencephalography, somatosensory evoked potentials, and biochemical serum markers of brain injury (for example, neuron-specific enolase), is recommended for outcome prognostication after cardiac arrest and therapeutic hypothermia [32]. A minimum observation time of 72 hours after cardiac arrest or a return to normothermia in hypothermic patients is required for evaluating individual patients [4]. Although completely predicting final outcomes for OHCA patients in the ED soon after arrival at the hospital is not possible, our developed decision-tree prediction model may contribute to preclinical quality assessment and help researchers analyze the effects of different postresuscitation strategies.

### Study limitations

The potential limitations of the current analysis are as follows. First, we did not consider the time-related factors to be potentially correlated with outcomes such as collapse time, time interval from collapse to ROSC, time interval from collapse to CPR initiation, and time interval from collapse to AED use. It could be difficult to recall the exact time-of-collapse events in emergency situations.

Second, we also did not analyze the model by using the variables of prehospital administration of adrenalin or advanced airway managements techniques performed by ELSTs because previous Japanese studies have reported that the prehospital use of adrenalin and advanced airway management do not increase the chance of survival and good functional outcomes after cardiac arrest among OHCA patients [33,34].

Third, the detailed in-hospital interventions were not evaluated. We assumed that OHCA patients received standard advanced life support according to the Japanese CPR guidelines [35], which are based on the 2000 and 2005 American Heart Association guidelines [36,37].

Fourth, it is not known whether our decision-tree model is valid for communities with other emergency care characteristics. It may be necessary for other countries to validate the present prediction model.

Fifth, unmeasured confounding factors might have influenced outcomes.

## Conclusions

On the basis of a decision-tree prediction model using four prehospital variables (shockable initial rhythm, age, witnessed arrest, and witnessed by EMS personnel), OHCA patients can be readily stratified into the four groups (good, moderately good, poor, and absolutely poor) that help predict both survival at 1 month and survival with favorable neurologic outcomes at 1 month. This simple prediction model may provide clinicians with a practical bedside tool for OHCA patient stratification in the ED.

## Key messages

• We developed a simple and generally applicable decision-tree prediction model for OHCA patients in the ED, by using a prospectively recorded nationwide Utstein-style Japanese database.

• The decision-tree model consists of four prehospital variables: shockable initial rhythm, age (younger than 70 years or not), witnessed arrest, and witnessed by EMS personnel.

• This model can readily stratify OHCA patients into groups at good, moderately good, poor, and absolutely poor for predicting 1-month survival and that with favorable neurologic outcome and may help guide clinician decision making and risk assessment for individual patients.

## Abbreviations

AED: automated external defibrillator; AUC: area under the receiver operating characteristic curve; CI: confidence interval; CPC: Cerebral Performance Category; CPR: cardiopulmonary resuscitation; ED: emergency department; ELST: emergency life-saving technician; EMS: emergency medical services; FDMA: Fire and Disaster Management Agency; ICU: intensive care unit; OHCA: out-of-hospital cardiac arrest; OR: odds ratio; RACA: German Resuscitation Registry Group developed the ROSC after cardiac arrest; ROSC: return of spontaneous circulation; SD: standard deviation.

## Competing interests

The authors declare that they have no competing interests.

## Authors' contributions

Yoshikazu Goto and Tetsuo Maeda designed the study. Yoshikazu Goto, Tetsuo Maeda, and Yumiko Goto conducted data cleaning. Yoshikazu Goto and Yumiko Goto analyzed the data. Yoshikazu Goto drafted the manuscript, and Yumiko Goto and Tetsuo Maeda contributed substantially to its revision. Yoshikazu Goto takes responsibility for the article as a whole. All authors approved the manuscript before submission.
